# Neuronal Ceroid Lipofuscinosis—Concepts, Classification, and Avenues for Therapy

**DOI:** 10.1111/cns.70261

**Published:** 2025-02-09

**Authors:** Yuheng Zhang, Bingying Du, Miaozhan Zou, Bo Peng, Yanxia Rao

**Affiliations:** ^1^ Department of Neurology, Zhongshan Hospital, Laboratory Animal Center Fudan University Shanghai China; ^2^ Children’s Hospital, Institute for Translational Brain Research, State Key Laboratory of Medical Neurobiology, MOE Frontiers Center for Brain Science, MOE Innovative Center for New Drug Development of Immune Inflammatory Diseases Fudan University Shanghai China; ^3^ Department of Neurology The First Affiliated Hospital of Naval Medical University Shanghai China

**Keywords:** enzyme replacement therapy, lysosomal storage disorders, microglial cell replacement therapies, neuronal ceroid lipofuscinosis

## Abstract

Neuronal ceroid lipofuscinosis (NCL) is a group of neurodegenerative lysosomal storage disorders characterized by excessive accumulation of lysosomal lipofuscin. Thirteen subtypes of NCL have been identified, each associated with distinct genes encoding various transmembrane proteins, secretory proteins, or lysosomal enzymes. Clinically, NCL manifests in infants through vision impairment, motor and cognitive dysfunctions, epilepsy, and premature death. The pathological complexity of NCL has hindered the development of effective clinical protocols. Current treatment modalities, including enzyme replacement therapy, pharmacological approaches, gene therapy, and stem cell therapy, have demonstrated limited efficacy. However, emerging evidence suggests a significant relationship between NCL and microglial cells, highlighting the potential of novel microglial cell replacement therapies. This review comprehensively examines the pathogenic genes associated with various NCL subtypes, elucidating their roles, clinical presentations, and corresponding mouse models. Especially, we thoroughly discuss the advances in the clinical study of potential therapeutics, which crucially calls for early diagnosis and treatment more than ever.

## Graphical abstract

This review comprehensively examined the pathogenic genes associated with various NCL subtypes, elucidating their roles, clinical presentations, corresponding mouse models, and the advances in clinical study of potential therapeutics. In particular, we clarified the potential of novel microglial cell replacement therapies in NCLs, providing hope for slowing or halting disease progression.

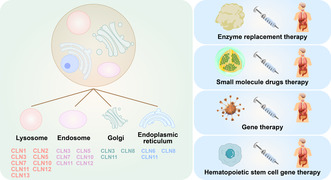

## 1 Introduction

Neuronal ceroid lipofuscinosis (NCL), also known as Batten disease, refers to a group of neurodegenerative lysosomal storage diseases (LSDs), which is characterized by the excessive accumulation of lysosomal lipofuscin in multiple tissue [1, 2]. NCL represents a complex and diverse group of neurodegenerative disorders with pathophysiology involving multiple genes and cellular processes. The most common forms of NCL affect infants and children, leading to progressive visual failure, seizures, motor dysfunction, cognitive decline, and ultimately premature death [3, 4, 5]. Less commonly, NCL can appear in early adulthood, presenting with early dementia, myoclonic seizures, movement abnormalities (ataxia, extrapyramidal), psychiatric disturbance, and visual failure [4, 5]. Although challenges remain in treating NCL, emerging therapies such as enzyme replacement therapy (ERT), pharmacological approaches, gene therapy, and stem cell therapy offer new hope for patients. This review aims to provide a comprehensive overview of the pathophysiology and current treatment strategies for NCL.

## 2 Overview of NCL

**FIGURE 1 |:**
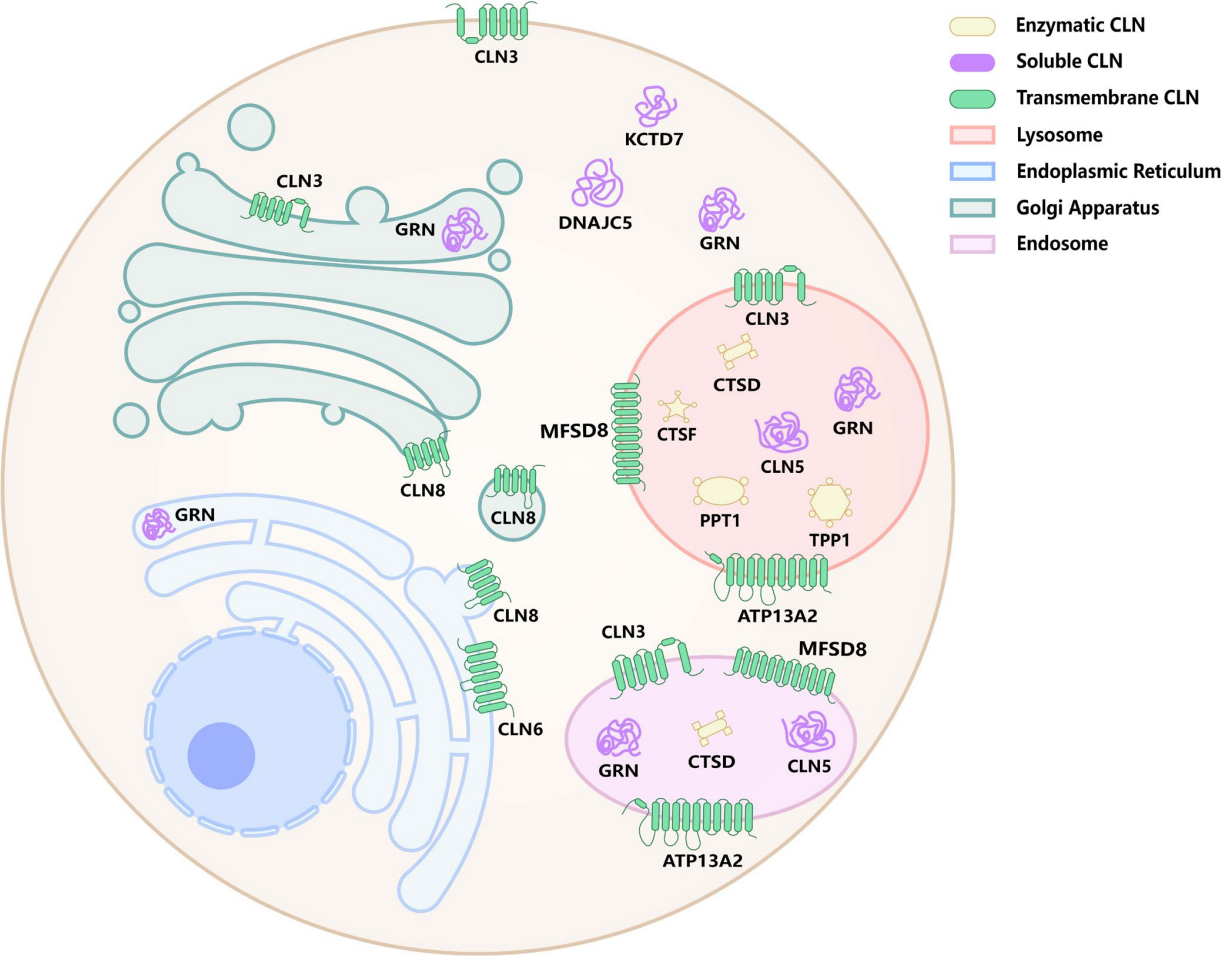
The locations of NCL proteins. *CLN1 (PPT1), CLN2 (TPP1), CLN10 (CTSD)*, and *CLN13 (CTSF)* encodes soluble NCL proteins with known enzymatic activity; *CLN4 (DNAJC5), CLN5, CLN11 (GRN)*, and *CLN14 (KCTD7)* encodes soluble proteins with one protein (encoded by *GRN*) localized to compartments in the secretory pathway; *CLN3, CLN6, CLN7 (MFSD8), CLN8*, and *CLN12 (ATP13A2)* encodes transmembrane proteins with different subcellular locations: CLN3 mainly localizes to the plasma membrane, Golgi, endosome and lysosome; CLN6 localize to the ER; MFSD8 mainly localize to the lysosome and endosome; CLN8 shuttles between the ER and the ERGIC compartment; ATP13A2 mainly localize to the membrane of lysosomes and late endosomes.

By 2015, 13 genes (*CLN1*/*PPT1*, *CLN2*/*TPP1*, *CLN3*, *CLN4*/*DNAJC5*, *CLN5*, *CLN6*, *CLN7/MFSD8, CLN8*, *CLN10*/*CTSD*, *CLN11*/*GRN*, *CLN12*/*ATP13A2*, *CLN13*/*CTSF*, and *CLN14*/*KCTD7*) have been identified as being associated with NCL. These include four genes encoding lysosomal enzymes (LEs) (*CLN1*/*PPT1*, *CLN2*/*TPP1*, *CLN10*/*CTSD*, and *CLN13*/*CTSF*), one encoding a soluble lysosomal protein (*CLN5*), one encoding a protein in the secretory pathway (*CLN11*/*GRN*), two encoding cytoplasmic proteins that also peripherally associate with membranes (*CLN4*/*DNAJC5*, *CLN14*/*KCTD7*), and five encoding transmembrane proteins with various subcellular locations (*CLN3*, *CLN6*, *CLN7/MFSD8, CLN8*, and *CLN12*/*ATP13A2*) [4]. The different properties and locations of these NCL proteins are illustrated in Figure 1.

One family, initially classified as CLN9 (ceroid lipofuscinosis neuronal) and associated with juvenile‐onset disease, has been reclassified as a variant of CLN5 for their mutation in the *CLN5* gene (c.694 T>C, p. Gln232X) [6]. Another patient previously proposed to represent CLN9 carries a homozygous *CLN2* mutation [7, 8]. Among the identified NCLs, CLN1, CLN2, and CLN3 are predicted to be the most frequently encountered [9]. All NCL genes are located on autosomes, with the disease typically inherited in a recessive manner, except for *CLN4*. In these recessive cases, deleterious mutations must be present in both alleles of the gene [10].

While most mutations in these genes are associated with a typical NCL disease phenotype, some result in variable disease onset, severity, and progression, causing distinct clinical phenotypes [4]. Based on the age of onset, NCL was classified into several types: congenital NCL (manifesting before or within a few weeks of birth), infantile NCL (INCL, onset from 6 to 24 months), late‐infantile NCL (LINCL, onset from 2 to 4 years), juvenile NCL (JNCL, onset from 4 to 15 years), and adult‐onset NCL (ANCL, onset from 15 to 50 years). At the same time, we should also note that many of the variant forms of the disease can occur at various times [3, 11]. The reported incidence of NCL varies by country, but it is estimated to affect approximately 2 out of every 100,000 live births [12].

### 2.1 CLN1

CLN1 disease, one of the most common forms of NCL, was first described in 1988. It is caused by biallelic loss‐of‐function variants in the *PPT1* gene, which encodes the lysosomal enzyme palmitoyl‐protein thioesterase 1 (PPT1) [13]. CLN1 typically manifests as INCL, with onset during childhood, typically between 6 and 24 months of age, leading to progressive blindness, cognitive decline, motor deficits, and seizures, resulting in death around 9 to 13 years of age (Table 1) [14, 15]. However, late‐infantile‐onset, juvenile‐onset, and adult‐onset forms of CLN1 have also been described [16].

**TABLE 1 |:** Summary of genotype and clinical characteristics in neuronal ceroid lipofuscinoses.

Disease name	Protein	Protein function	Human on‐set	Human clinical manifestation
CLN1	PPT1	In lysosomes, the protein cleaves the thioester linkage of the fatty acid palmitate‐to‐cysteine residues of palmitoylated proteins (constituents of ceroid), a step essential for the degradation within lysosomes	6–24 months	It manifests clinically as INCL, with progressive blindness, cognitive decline, motor deficits, and seizures. It has an invariably fatal outcome by 9–13 years of age. Late‐infantile‐onset, juvenile‐onset, and adult‐onset forms of CLN1 have also been described
CLN2	TPP1	TPP1 cleaves tripeptides from the amino terminus of small polypeptides undergoing degradation in the lysosomes. It also has weak endopeptidase activity, and is targeted to lysosomes via the M6P receptor pathway	4–8 years	Patients with CLN2 disease manifests with motor decline, speech delay, and seizures, and typically with death occurring between 7 and 15 years of age
CLN3	CLN3 Lysosomal/Endosomal transmembrane protein	CLN3 is required for the efflux of GPDs from the lysosome so that GPDs can be degraded by glycerophosphodiesterase (GDE) in the cytosol, and it is a vesicular trafficking hub connecting the Golgi and lysosome compartments. Loss of CLN3 has been shown to affect PPT1, TPP1, CLN5, and CTSD	4–8 years	Patients with CLN3 disease have an onset of symptoms with seizures, visual deficits, and motor decline, followed by cognitive decline, and typically fatal by 20–30 years of age
CLN4	CSPα	The J‐domain of CSPα can activate the ATPase activity of HSP70 and position HSP70 toward specific clients such as CSPα	Adult (above 20 years)	Disease presenting with progressive myoclonus epilepsy
CLN5	CLN5 intracellular trafficking protein	One of the major palmitoyl thioesterases in neuronal cells	4–7 years	Clinical features of vLINCL with motor clumsiness, followed by progressive visual failure resulting in blindness, as well as motor and mental deterioration. Seizures and death occur between 14 and 36 years of age
CLN6	CLN6 transmembrane ER protein	CLN6 and CLN8 are obligate partners for the recruitment of newly synthesized lysosomal enzymes at the ER	From 18 months to 8 years	Patients with classic CLN6 disease have an onset of symptoms with features of blindness, seizures, and cognitive decline
CLN7	MFSD8	CLN7 is located in lysosomes and endosomes and is considered as an endolysosomal chloride channel	Juvenile and adult	Patients suffer from seizures, developmental regression, loss of vision, and premature death
CLN8	CLN8 transmembrane ER and ERGIC protein	CLN8 mediates transfer of newly synthesized lysosomal enzymes from the ER to the Golgi complex	2–16 years	Manifests with seizure, followed by progressive vision loss and developmental regression
CLN10	CTSD	CTSD is one of the major lysosomal hydrolases for the maintenance of cellular proteostasis by degrading substrates of endocytosis, phagocytosis, and autophagy, such as the amyloid precursor, α‐synuclein, and huntingtin	From infancy to adulthood	Patients present with progressive cognitive decline, loss of speech, visual dysfunction, and loss of motor functions
CLN11	PGRN	Secreted PGRN is processed to granulin peptides by several different proteases. PGRN can facilitate neuronal uptake and lysosomal delivery of PSAP, and lysosomal targeting of microglia‐derived PSAP in neurons relies in part on PGRN and sortilin	From 5 to 25 years	Most patients present with symptoms of epilepsy (seizures), progressive vision impairment, ataxia, and cognitive decline
CLN12	ATP13A2	It functions as a late endolysosomal transporter, regulating ion homeostasis (Ca2+, Mn2+, Zn2+, etc.), lipid homeostasis, and autophagy‐lysosomal pathway	Patients have an onset of gait disturbance at approximately 13 years of age, followed by dysphagia, motor dysfunction, and cognitive impairment in their 20s	CLN12 disease typically presents with motor dysfunction and cognitive decline
CLN13	CTSF	CTSF is responsible for the lysosomal processing of wild‐type LIMP‐2	After 20 years	Patients with CLN13 disease develop progressive behavioral abnormalities and dementia after 20 years of age
CLN14	KCTD7	The CRL3‐KCTD7 complex recruits and degrades CLN5 via the ubiquitin‐proteasome pathway. A KCTD7 mutant mouse was generated through a deletion of exon 2	14 months	Patients with KCTD7 mutations have an onset of seizure at 14 months of age, followed by delayed development with subsequent progressive regression, and most patients would die between 1.5–21 years of age

Abbreviations: CLN, neuronal ceroid lipofuscinosis type; CRL3, CUL3‐RING E3 ubiquitin ligase; CSPα, Cysteine‐string protein alpha; CTSD, cathepsin D; CTSF, cathepsin F; ER, endoplasmic reticulum; GDE, glycerophosphodiesterase; GPDs, glycerophosphodiesters; Hsp70, heat shock protein 70; INCL, infantile neuronal ceroid lipofuscinosis; KCTD7, potassium channel tetramerization domain‐containing protein 7; LIMP‐2, lysosomal integral membrane protein type‐2; M6P, mannose 6‐phosphate; MFSD8, major facilitator superfamily domain–containing 8; PGRN, progranulin; PPT1, palmitoyl protein thioesterase 1; PSAP, prosaposin; TPP1, tripeptidyl peptidase 1; vLINCL, variant late‐infantile neuronal ceroid lipofuscinosis.

The nascent PPT1 polypeptide comprises 306 amino acids and migrates as a 37/35 kDa doublet in its mature form [17]. PPT1 is primarily transported to lysosomes via the mannose 6‐phosphate (M6P) receptor‐mediated pathway [18]. In the lysosome, PPT1 cleaves the thioester linkage of the fatty acid palmitate‐to‐cysteine residues of palmitoylated proteins (constituents of ceroid), a crucial step for their degradation within lysosomes [13]. In addition to lysosomes, PPT1 localized in synaptosomes and synaptic vesicles in neurons with significant secretion of the enzyme observed [19, 20].

PPT1 is implicated in synaptic growth, lipid metabolism, endosomal/synaptosomal trafficking, and intracellular signaling. It is essential for proper neuronal cell fates and organization, as well as for establishing the local environment for proper axon guidance and fasciculation [21]. Post‐mortem brain samples from CLN1 patients revealed altered phospholipid content, with increased phosphatidylcholine (PC) and decreased phosphatidylethanolamine (PE) and phosphatidylserine (PS) [22]. PPT1 also regulates tumor necrosis factor (TNF)‐induced pathways through depalmitoylation, with its deficiency conferring resistance to TNF‐induced apoptosis [23].

There are two different mouse models of CLN1, generated by different mutations in the *Ppt1* gene. One was generated via the insertion of a neomycin resistance gene (neo) cassette into exon 9 (PPT1‐deficient mice) [24]; the other was generated by Cre/loxp‐mediated deletion of exon 4 (${\text{Ppt1}}^{\text{Δex4}}$ mice) (Table 2) [25]. PPT1‐deficient mice exhibit frequent myoclonic jerks, seizures, lack of grooming, and progressive gait abnormalities (Table 2) [24]. Both PPT1‐deficient mice and ${\text{Ppt1}}^{\text{Δex4}}$ mice display significant reduction in lifespan (approximately 210–300 days for PPT1‐deficient mice and 200 days for ${\text{Ppt1}}^{\text{Δex4}}$ mice) [24, 25]. Both CLN1 models show a significant reduction in brain mass (approximately 50%) [24, 25], a progressive degeneration of various retinal cell types, as well as altered lysosomal pH [26]. Remarkably, neuronal loss has been observed in the cortex, thalamus, cerebellum (specifically Purkinje cell dropout), and hippocampus (including neurons and GABAergic interneurons) before 6 months of age [24, 25]. Early loss of thalamic neurons occurs at 3 months in the dorsal lateral geniculate nucleus, later in the medial geniculate nucleus and in the ventral posterior nucleus. This precedes neuron loss in other areas such as the cortex, potentially contributing to the onset of visual deficits and seizures [27].

**TABLE 2 |:** Summary of mouse model construction, genotype, and phenotypes of neuronal ceroid lipofuscinoses.

Disease name	Mouse model	Mouse on‐set	Mouse behavior
CLN1	The first was generated via insertion of a neomycin resistance gene (neo) cassette into exon 9 (PPT1‐deficient mice); The second was generated by Cre/lox‐mediated deletion of exon 4 (*Ppt1* ^*Δex4*^)	About 4 months	Mice exhibit a progressive onset of seizures, abnormal clasping behavior, loss of motor coordination, and vision loss
CLN2	Mouse model was generated by inserting a neo cassette into intron 11 of *Tpp1*, combined with a missense Arg446His mutation in exon 11 (neoinsArg446His) (CLN2‐deficient mice)	About 7 weeks	Mice exhibit tremor and progressive motor dysfunction at about 7 weeks of age, followed by seizures
CLN3	*Cln3* ^*Δex7/8*^ mice (*Cln3* ^*Δex7/8*^ mice) have an identical genomic DNA deletion of a common human *CLN3* mutation introduced into the murine *Cln3* homologue	2–4 months	CLN3Δex7/8 mice exhibit a delayed but progressive onset of visual failure, learning/memory impairment, and motor dysfunction that correlates
CLN4	Isolated genomic clones that contained the first coding exon of the mouse *Dnajc5* gene and constructed a targeting vector for homologous recombination, then electroporated the targeting vector into embryonic stem cells, selected homologous recombinants to generate CSPα KO mice (CSPα KO mice)	2–4 weeks	The phenotype of CSPα KO mice developed progressive loss of synaptic function and neurodegeneration with muscle weakness and sensorimotor disorder
CLN5	A mouse model for CLN5 was generated by targeted deletion of exon 3 of the mice *Cln5* gene, which results in a frameshift and a premature stop codon in exon 4 (*Cln5* ^*−/−*^ mice)	Severe loss of vision at an average of 21 weeks of age; have symptoms of progressive motor abnormalities or development of seizures at 8 months of age	Loss of vision, symptoms of progressive motor abnormalities or development of seizures
CLN6	*Cln6* ^*nclf*^ mice was subsequently confirmed to have a single bp insertion in exon 4 that results in a frameshift mutation (*Cln6* ^*nclf*^ mice)	8–9 months	CLN6 nclf mice show a decline in rotarod performance, and performed poorly at the Morris water maze starting. More than half of CLN6 nclf mice died by 14–15 months of age
CLN7	Deletion of *Cln7* exon 2 leads to complete loss of CLN7 in *Cln7* KO mice (*Cln7* KO mice)	4–6 months	*Cln7* KO mice performed poorly at the visual water maze starting. The lifespan of *Cln7* KO mice is about 8–10 months
CLN8	The classic mouse model for CLN8 disease is the motor neuron degeneration mice, also known as *Cln8* ^*mnd*^, with a 1 bp insertion that causes a frameshift and truncated protein (*Cln8* ^*mnd*^ mice)	4–6 months	*Cln8* ^*mnd*^ mice exhibit impaired contextual and cued memory in the fear‐conditioning test, and more aggressive in the resident intruder test. Memory and spatial learning deficits and motor deficits are observed at 6 months of age. The lifespan of *Cln8* ^*mnd*^ mice is approximately 10 months
CLN10	The neo cassette was inserted into exon4 of the *Ctsd* gene to generate CTSD‐deficient mice (CTSD‐deficient mice)	About 2 weeks	These mice stop thriving in the third week with manifestations of anorexia, seizures, and progressive retinal atrophy, which leads to blindness, and die at approximately 26 days of age
CLN11	*Grn* ^*R493X/R493X*^ mice harbor a GRN R504X nonsense mutation (*Grn* ^*R493X/R493X*^ mice) Grn‐cKO mice is generated by deletion of exon 2–4 (Grn‐cKO mice) PGRN‐deficient mice is generated by deletion of exon 1–4 (PGRN‐deficient mice)	3–18 months	Displayed less anxiety at 3 months of age, developed depression‐like behavior at 4 months of age, had abnormal olfactory function at 18 months of age which would cause impaired social behavior in the social recognition test, and also showed spatial learning and memory deficits at 18 months of age
CLN12	Exons 12–15 and part of exon 16 of *Atp13a2* gene were replaced by the neo cassette to generate *Atp13a2* ^*−/−*^ mice (*Atp13a2* ^*−/−*^ mice)	20–29 months	Mice exhibited significant impairments in motor tests
CLN13	Exons 7–9 of *Ctsf* gene were deleted by homologous recombination with a targeting vector containing a neo cassette to generate *Ctsf* ^*−/−*^ mice (*Ctsf* ^*−/−*^ mice)	12–15 months	Mice displayed significant weight loss and a decline in motor coordination of the hind limbs at 12 months of age, followed by clear irregularities in the gait at 15 months of age. Most CTSF^−/−^ mice die at about 18 months of age
CLN14	*Kctd7*‐deficient mice was generated through a deletion of exon 2 (*Kctd7*‐deficient mice)	2 months	These mice exhibited seizures and myoclonic jerks at 2 months of age, and displayed defects in gait at of the same age, characterized by decreased print area, swing speed, and speed for each of the four paws

Abbreviations: CLN, neuronal ceroid lipofuscinosis type; CSPα, cysteine‐string protein alpha; CTSD, cathepsin D; CTSF, cathepsin F; DNAJC5, DnaJ heat shock protein family (Hsp40) member C5; GRN, granulin; KCTD7, potassium channel tetramerization domain‐containing protein 7; PPT1, palmitoyl protein thioesterase 1; PTC, premature termination codons; TPP1, tripeptidyl peptidase 1.

### 2.2 CLN2

Patients with CLN2 disease manifest with motor decline, speech delay, and seizures between 4 and 8 years of age. Most patients with CLN2 died between 7 and 15 years of age (Table 1) [28]. CLN2 was classified as LINCL; recently, both infantile‐onset and juvenile‐onset forms of CLN2 have also been described. A rare variant of CLN2, autosomal‐recessive spinocerebellar ataxia 7 (SCAR7), caused by variants in tripeptidyl peptidase 1 (*TPP1*) gene, has an onset of symptoms after 11 years of age [11, 28, 29].

TPP1 is a lysosomal exopeptidase. Upon acidification, TPP1 is processed from an inactive proenzyme to a 46 kDa active protein. The mature enzyme cleaves tripeptides from the amino terminus of small polypeptides undergoing degradation in the lysosomes. It also exhibits weak endopeptidase activity and is targeted to lysosomes via the M6P receptor pathway [30, 31]. The pro‐apoptotic BCL‐2 family member Bid has been identified as a substrate for TPP1, TPP1‐induced cleavage of Bid regulates TNF‐induced apoptosis. Apoptosis induced by TNF and related ligands is strongly inhibited in CLN2 deficient cells [32]. However, the substrates of TPP1 are not well characterized, and the pathological mechanisms underlying CLN2 remain unclear [30].

CLN2‐deficient mice were generated by inserting a neo cassette into intron 11 of the *Tpp1* gene, combined with a missense Arg446His mutation in exon 11 (neoinsArg446His), completely disrupting the protein's structure (Table 2) [33]. CLN2‐deficient mice show no obvious phenotype at birth, but autofluorescent storage appears as early as 48 days, accompanied by progressive neuropathology [33]. By seven weeks of age, the mice exhibit tremors and motor dysfunction, followed by seizures, with a mean survival of approximately 138 days. (Table 2) [33].

### 2.3 CLN3

Patients with CLN3 have an onset of symptoms in early childhood (ages 4–8), such as seizures, visual impairment, and motor decline, followed by cognitive deterioration. This disease is typically fatal by 20–30 years of age (Table 1) [11, 34].

The *CLN3* gene encodes a protein of 438 amino acids with six transmembrane domains, primarily found in late endosomes and lysosomes [35]. It has been shown to localize to other cellular structures, including the plasma membrane, Golgi, mitochondria, nucleus, and synapses [35, 36]. Glycerophosphodiesters (GPDs) are terminal products of phospholipid degradation in the lysosome. Studies have shown an accumulation of GPDs, including glycerophosphoinositol (GPI), glycerophosphoethanolamine (GPE), glycerophosphocholine (GPC), and glycerophosphoserine (GPS) in the lysosomes of CLN3‐deficient mice, suggesting that CLN3 may help export GPDs from lysosomes for degradation in the cytosol [37, 38]. CLN3 is essential for proper vesicular trafficking between the Golgi and lysosomes. It interacts with several endolysosomal trafficking proteins, including the cation‐independent mannose 6‐phosphate receptor (CI‐M6PR), which is crucial for targeting LEs. Significantly, CLN3 is not a Golgi‐resident protein, but rather the newly synthesized protein transits through the Golgi to reach the lysosomes [39]. Retromer is required for the efficient endosome‐to‐TGN (trans‐Golgi network) trafficking of CI‐M6PR and lysosomal sorting receptor, sortilin. CLN3 interacts with retromer and modulates the sortilin–retromer interaction, so mutations in *CLN3* result in mis‐trafficking of CI‐M6PR from the TGN, leading to its degradation in lysosomes, mis‐sorting of lysosomal enzyme and defective autophagy [39, 40, 41]. On the other hand, overexpression of CLN3 promotes the formation of multiple lysosomal tubules, aiding in autophagy and CI‐M6PR‐dependent processes [39]. The loss of CLN3 impacts other enzymes and proteins, such as PPT1, TPP1, CLN5, and CTSD [42, 43]. Cells lacking CLN3 are more prone to apoptosis and cell death, which may relate to disrupted Golgi function and ceramide accumulation [3, 44]. These changes are linked to alterations in autophagy, with an accumulation of autophagic vacuoles due to impaired endosome‐lysosome function [3, 44]. Additionally, CLN3‐deficient neurons have an abnormal cytoskeleton and disrupted turnover of Na^+^/K^+^‐ATPase at the plasma membrane, indicating a role for CLN3 in cytoskeletal organization and endocytosis [45].

${\text{Cln3}}^{\mathrm{\Delta ex}7/8}\;\text{mice}$, which mimic a common human *CLN3* mutation, are generated by introducing a mutation into the murine *Cln3* homologue, resulting in a frameshift mutation and a premature termination codon (PTC) [46, 47] (Table 2). ${\text{Cln3}}^{\mathrm{\Delta ex}7/8}\;\text{mice}$ exhibit measurable motor deficits at 2 months of age and significant deficits in sensorimotor coordination at 3 months of age [34]. By 4 months, female rather than male ${\text{Cln3}}^{\mathrm{\Delta ex}7/8}\;\text{mice}$ have a significantly lower forelimb grip strength [34]. Later on, ${\text{Cln3}}^{\mathrm{\Delta ex}7/8}\;\text{mice}$ develop a progressive visual failure, learning/memory impairment, and motor dysfunction, which correlates with noticeable neuron loss in several brain regions, including the retina, striatum, thalamus, cortex, cerebellum and substantia nigra (Table 2) [3, 47]. Compared with human patients with the *CLN3* mutation, these mice have a relatively longer lifespan, 20% die by 1 year of age [47].

### 2.4 CLN4

CLN4 originally referred to all adult‐onset forms of NCL, including autosomal‐dominant ANCL (Parry disease) and autosomal‐recessive ANCL (Kufs disease). Now, we know that Kufs disease with progressive myoclonus epilepsy (type A) is associated with mutations in the *CLN6* gene, while Kufs disease with dementia and motor disturbances (type B) is linked to mutations in the *CTSF* gene (also known as *CLN13*) [10, 48, 49]. Parry disease, now classified as CLN4, is caused by mutations in the DnaJ heat shock protein family (Hsp40) Member C5 (*DNAJC5*) gene, which encodes cysteine‐string protein alpha (CSPα) [10]. Patients with CLN4 disease display various symptoms, such as seizures, myoclonus, and cognitive decline, and usually die within 20 years of onset (Table 1) [50].

The *DNAJC5* gene has three subtypes: *DNAJC5a*, *DNAJC5b*, and *DNAJC5*, encoding CSPα, β, and γ, respectively [51]. CSPα is primarily expressed in neurons and is known as a cysteine protein with 12 to 14 cysteine residues. CSPα is a member of the HSP40 (DnaJ) family, which plays a crucial role in the assembly and disassembly of macromolecular complexes [52]. This family is also known as the J‐domain protein (JDP) family, which is an important partner of HSP70 (DnaK) [53]. The J‐domain of CSPα can activate the ATPase activity of HSP70, directing it toward specific clients like CSPα [52, 54, 55, 56]. JDP also contributes to the functional diversity of the HSP70 chaperone system [54]. Substrates of the CSPα–HSP70 chaperone complex include synaptosomal‐associated protein, 25 kDa (SNAP‐25), and GTPase dynamin 1, which are necessary for synaptic vesicle fusion and fission, regulating exocytosis and endocytosis of synaptic vesicles [57]. In cooperation with chaperones, CSPα ensures the function and survival of synapses [52].

CSPα mice appear normal at birth but develop progressive synaptic dysfunction, muscle weakness, and sensorimotor disorder by 2–4 weeks of age (Table 2) [51]. These mice begin to die at 3 weeks of age, with most dying by 2 months and none surviving beyond 3 months [51]. Alpha‐synuclein (α‐syn), a major component of Lewy bodies in Parkinson's disease (PD), aggregates early at the synapse and can spread toxicity through cell‐to‐cell transmission [58, 59, 60, 61]. Membrane targeting of CSPα requires palmitoylation for membrane targeting, and CSPα palmitoylation deficiency reduces. α‐syn secretion [58]. CSPα also inhibits the ubiquitination, phosphorylation, and aggregation of α‐syn. Lack of CSPα results in α‐syn aggregation and associated neurotoxicity [62]. Targeting CSPα and relevant chaperones may provide therapeutic potential to restore synaptic function in early‐stage PD [52].

### 2.5 CLN5

CLN5 subtype was first described in Finland, also called Finnish variant LINCL (vLINCL). It has later been identified in various ethnic populations [11, 63]. The precursor CLN5 protein, composed of 407 amino acids, localized in the endolysosomal compartment, is membrane‐located and cleaved by signal peptide peptidase (SPP) and its homologs SPP‐like proteases (SPPL), to become soluble and active CLN5 [63, 64]. Patients with CLN5 disease experience delayed psychomotor development and visual failure. These symptoms typically begin between ages 4 and 7 with motor clumsiness, followed by progressive visual failure resulting in blindness, as well as motor and mental deterioration. Seizures and death occur between 14 and 36 years of age (Table 1) [11, 65].

CLN5 is a member of the palmitoyl thioesterase family, regulating cellular protein S‐palmitoylation, and is one of the major palmitoyl thioesterases in neuronal cells [63]. Palmitoylation, a classical kind of lipid modification, regulates neuronal protein trafficking and function. The reversible nature of palmitoylation allows proteins to shuttle between intracellular compartments [66]. The reversible attachment of a palmitic acid onto a cysteine residue via a thioester linkage (S‐palmitoylation) is crucial for the assembly and compartmentalization of proteins, which is essential for neuronal development and synaptic plasticity. Disturbed S‐depalmitoylation may impair neuronal function [66]. CLN5‐deficient neuronal progenitor cells showed reduced thioesterase activity, suggesting that CLN5 may also play some roles in S‐depalmitoylation [63]. Interestingly, a recent publication by Medoh et al. reported that the Batten disease gene product CLN5 is the elusive bis(monoacylglycero)phosphate (BMP) synthase. Lysosomes critically rely on BMP to stimulate lipid catabolism, cholesterol homeostasis, and lysosomal function [67]. CLN3 and CLN5 form a late endolysosomal complex that regulates endocytic trafficking. This complex can recycle sortilin, a lysosomal enzyme sorting receptor, from endosomes back to TGN [68]. Loss of CLN3 has been shown to affect several lysosomal proteins, including PPT1, TPP1, CLN5, and CTSD. Similarly, the loss of CLN5 impacts the functions of multiple lysosomal proteins, such as PPT1, TPP1, CLN3, CLN6, CLN8, CTSD, and KCTD7 (CLN14). For example, CLN5 modulates the trafficking of TPP1 and CTSD to lysosomes [43, 69].

A mouse model for CLN5 was generated by deleting exon 3 of the murine *Cln5* gene, which results in a frameshift and a premature stop codon in exon 4 [65]. *Cln5* ^−/−^ mice show progressive visual failure beginning around 13 weeks of age and severe loss of vision at an average of 21 weeks of age (Table 2). However, they do not demonstrate progressive motor abnormalities or seizures until 8 months of age [65]. *Cln5* ^−/−^ mice exhibit progressive cortical neuron loss and synaptic pathology since 4 months of age, with neuron loss becoming evident by 12 months of age [70].

### 2.6 CLN6

CLN6 disease, also known as Kufs disease type A. Patients typically exhibit symptoms in early childhood (between 18 months and 8 years), including blindness, seizures, and cognitive decline (Table 1). This disease is fatal by age 20. Non‐classic CLN6 disease has been identified in teenagers, showing a later of symptoms [3, 4, 71, 72, 73].

CLN6 is a 30 kDa protein with 311 amino acids that is located in the endoplasmic reticulum (ER). Mutations in the *CLN6* gene lead to decreased synthesis and storage [74]. LEs are synthesized in the ER and transferred to the endolysosomal system via the secretory route. This process is mediated by CLN8, a multipass membrane protein that forms homodimers and localizes in the ER and the ER‐Golgi intermediate compartment (ERGIC) [75]. CLN8 interacts with newly synthesized LEs in the ER, transferring them to the Golgi via coat protein complex II (COPII) vesicles, and recycling back to the ER via COPI vesicles. CLN6, which also resides in the ER, partners with CLN8 to recruit newly synthesized LEs. Unlike CLN8, CLN6 remains in the ER, presumably to facilitate additional enzyme recruitment cycles. The interaction of CLN6 with these enzymes is crucial for their efficient ER export and subsequent lysosomal function. CLN6 deficiency results in inefficient ER export of LEs and diminished levels of the enzymes at the lysosome [76]. In CLN6‐deficiency fibroblasts and neurons, the mitochondrial enzyme manganese‐dependent superoxide dismutase (MnSOD, SOD2) is increased, likely due to oxidative stress and pro‐inflammatory cytokine production [77]. Additionally, CLN6 interacts with collapsing response mediator protein‐2 (CRMP‐2), involved in microtubule assembly and cytoskeletal dynamics during axonal growth, which is correlated with reduced neuronal growth and maturation [78].

CLN6 transcripts are expressed in all adult human and mice tissues, with varying levels in different brain regions [71, 74]. Cellularly, *Cln6* mRNA is prominently expressed in the brain's cortical layers II–VI, the Purkinje cell layer, dentate gyrus, and the hippocampal pyramidal cell layer of the CA1 region [79]. In *Cln6* ^*nclf*^ mice, a single base pair insertion in exon 4 that results in a frameshift mutation, similar to that in human patients with CLN6 disease, results in a novel 36 kDa gene product [71]. The half‐life of CLN6 is over 30 h in wild‐type, whereas it is about 10 h in *Cln6* ^*nclf*^ mice, suggesting that the rate of synthesis and the stability of mutant CLN6 proteins are reduced [80]. *Cln6* ^*nclf*^ mice show a decline in rotarod performance by 8 months and poor performance in the Morris water maze by 9 months [72]. Over half of these *Cln6* ^*nclf*^ mice died by 14–15 months (Table 2) [72].

### 2.7 CLN7

The CLN7 subtype was first described in Turkish patients and is associated with the variant late‐infantile NCL (vLINCL). Later, this type of NCL has been reported in juvenile and adult patients in various countries [11, 81]. Turkish CLN7 disease typically begins between 2 and 7 years of age with symptoms including seizures, developmental regression, vision loss, and premature death before age 20 (Table 1) [11, 81, 82].

The human CLN7 protein containing 518 amino acids is a major facilitator superfamily (MFS) protein, also known as MFS domain‐containing 8 (MFSD8) [81]. CLN7 mRNA is more abundant in neurons than in astrocytes and microglia. CLN7 is located in lysosomes and endosomes, this localization of CLN7 is mainly determined by an N‐terminal dileucine motif, which specifically binds to the heterotetrameric adaptor AP‐1(origin from TGN), and is not impaired by the presence of pathogenic missense mutations or after genetic ablation of the N‐glycans [83]. CLN7 functions as an endolysosomal chloride channel, promoting lysosomal calcium release through transient receptor potential mucolipin 1 (TRPML1), which activates CaM and aids lysosomal fusion [84]. CLN7's role in lysosomal enzyme trafficking may involve functioning as a chloride channel [84, 85]. Its loss leads to a decrease in chloride permeability, affects enzymes such as PPT1, TPP1, CLN5, and CTSD, reducing their amounts in lysosomes and altering their activity [43, 84]. Loss of CLN7 function reduces the amounts of PPT1, CLN5, and CTSD in lysosomes and decreases the amount of TPP1 in human urine [86, 87].

In *Cln7* KO mice, retinal degeneration and poor visual water maze performance are observed at 4 to 6 months of age, with behavioral deficits noted in rotarod, open field, and marble burying tests by 6 months. These mice have a lifespan of about 8–10 months (Table 2) [84, 88, 89].

### 2.8 CLN8

CLN8 disease manifests with seizure onset at about 5 years of age (range: 2–16 years of age), followed by progressive vision loss and developmental regression (Table 1) [11, 90, 91]. *CLN8* mutations result in two main phenotypes: one is Northern epilepsy (NE), a progressive epilepsy with mental retardation (EPMR) prevalent in Finland; the other one is late‐infantile NCL, primarily reported in Turkey, characterized by severe cognitive impairment and loss of mobility within 2–4 years [91, 92, 93].

*CLN8* encodes a 286 amino acid transmembrane protein and is primarily localized in the ER, with partial localization in the ERGIC. CLN8 mediates the transfer of newly synthesized LEs from the ER to the Golgi complex [76, 94]. The classic mouse model for CLN8 disease is *Cln8* ^mnd^, with a 1 base pair insertion that causes a frameshift and production of truncated protein [95].

*Cln8* ^*mnd*^ mice exhibit impaired contextual and cued memory in the fear‐conditioning test and are more aggressive in the resident intruder test by 4 to 5 months of age. These mice showed impaired spatial learning and memory by 6 months of age and motor deficits at the same age, with a lifespan of approximately 10 months (Table 2) [96, 97, 98]. Interestingly, female *Cln8* ^*mnd*^ mice performed in a water maze test and have shorter lifespan [98]. PP2A is one of the major serine/threonine phosphatases in cells. The phosphorylation levels of several substrates of PP2A, such as Akt, S6 kinase, and GSK3β, are decreased in patients with CLN8 disease, suggesting increased PP2A activity in CLN8 patients [99]. Ceramides are known to bind to and influence the activity of PP2A, and the ceramide levels are reduced by 60% in CLN8 patients [99]. Both CLN6 and CLN8 play an essential role in recruiting LEs to the membrane of ER [76]. Loss of CLN6 and CLN8 impairs the localization and activity of multiple enzymes, such as PPT1 [43].

### 2.9 CLN10

CLN10 is classified as congenital NCL. CLN10 manifests variable onset from infancy to adulthood. CLN10 is caused by dysfunction of Cathepsin D (CTSD), leading to progressive cognitive decline, loss of speech, visual dysfunction, and motor function loss (Table 1) [11, 100]. CLN10 shows the most severe clinical phenotype among NCL subtypes. The complete loss of CTSD activity causes early death in newborns [101].

CTSD, an aspartic protease, is expressed in the lysosome of all kinds of cells in the body, with high expression levels in the central nervous system (CNS) [102, 103, 104]. CTSD is synthesized on the rough endoplasmic reticulum (RER) as a pre‐pro‐enzyme (zymogen) and undergoes several proteolytic cleavages during translation, including the removal of a 20 amino‐acid signal peptide. After being transported to the Golgi apparatus, the 52 kDa pro‐CTSD is tagged with two M6P residues and targeted to lysosomes. In the endosome, pro‐CTSD loses a 44‐amino acid inhibitory pro‐peptide to become an enzymatically active 48 kDa single‐chain intermediate. This intermediate then forms the mature double‐chain form in the lysosome. During the process of autophagy, autophagosomes are deprived of proteolytic enzymes and fuse with hydrolase‐rich lysosomes to degrade their proteins. This process relies on a family of lysosomal proteases called cathepsins, including the cysteine proteinases CTSB (Cathepsin B), CTSF, CTSL (Cathepsin L), and the aspartyl protease CTSD [103, 105]. CTSD is one of the major lysosomal hydrolases for the maintenance of cellular proteostasis by degrading substrates from endocytosis, phagocytosis, and autophagy. These substrates include amyloid precursor, α‐synuclein, and huntingtin, which are implicated in neurodegenerative diseases [102, 103, 105].

The neo cassette was inserted into exon4 of the *Ctsd* gene to generate CTSD‐deficient mice [106]. CTSD‐deficient mice develop normally for the first 2 weeks and show severe symptoms by the third week and die at approximately 26 days of age, with signs of anorexia, seizures, and progressive retinal atrophy (Table 2) [106, 107]. CTSD could be regulated by several CLN proteins. For example, loss of PPT1, TPP1, and GRN(as known as CLN11) increases CTSD levels in tissues [108, 109, 110, 111], while loss of CLN3, CLN5, CLN6, CLN8, or ATP13A2(CLN12) reduces them [68, 76, 112].

### 2.10 CLN11

CLN11, also known as Granulin (GRN), was first described in 2012 in two siblings with ANCL. Symptoms can appear between 5 and 25 years of age, presenting as either juvenile‐onset (NCL‐like) or adult‐onset (frontotemporal dementia [FTD]‐like) phenotype [113, 114, 115, 116]. Heterozygous *GRN* mutations lead to progranulin haploinsufficiency and cause FTD in the elderly population, while homozygous *GRN* mutations cause juvenile‐onset or adult‐onset NCL [115]. Most patients with homozygous mutation present with various symptoms, including epilepsy (seizures), progressive vision impairment, ataxia, and cognitive decline (Table 1) [116]. Most patients have a relatively long lifespan (over 30 years old), but experience severe quality of life impairment [116]. There was a case reported that one female patient displayed recurrent seizures by 14 years of age. Her elder sister developed seizures by age 8 years old and died at 16 years old [113].

Progranulin (PGRN), encoded by the *CLN11*/*GRN* gene, is found in various subcellular organelles, including the ER, Golgi apparatus, endosome, and lysosomes. It also can be secreted outside the cells [117]. In the CNS, PGRN is highly expressed in microglia and, to a lesser extent, in neurons and other cell types [117, 118]. PGRN is synthesized in the ER lumen, and ER exit requires interaction with prosaposin (PSAP), which in turn binds to Surf4, an ER receptor that promotes loading of lumenal cargos into COPII vesicles destined for the Golgi complex [119]. PGRN can then follow the secretory pathway to migrate to the endolysosomal compartment. PGRN, composed of 593 amino acids, is processed to various 6 kDa granulin peptides by several cleavage proteases, including matrix metalloproteinases 9 and 14, a disintegrin and metalloproteinase with thrombospondin motif 7 (ADAMTS‐7), neutrophil elastase, proteinase 3, and intracellular by cathepsin L (Cat L) [120, 121]. These products of PGRN are defined as GRN peptides, including P (AA18‐49), G (AA61‐112), F (AA126‐178), B (AA208‐260), A (AA284‐335), C (AA336‐416), D(AA444‐495), and E (AA521‐572) [122]. Their peptides are involved in lysosomal proteolysis and lipid catabolism (Table 1) [123, 124]. PGRN, which also binds to bis monoacylglycero phosphate (BMP) and β‐Glucocerebrosidase (GCase), plays a role in lysosomal lipid degradation, including Glucosylsphingosine (GlcSph), the primary substrate of GCase [125, 126].

The human *GRN* gene, located on chromosome 17, contains 12 protein‐coding exons, while the mouse *Grn* gene, located on chromosome 11 spans approximately 6.3 kbp and contains 13 exons [127]. ${\mathrm{Grn}}^{R493X/R493X}\;\text{mice}$ harbor a *Grn* R504X nonsense mutation analogous to the human arginine 493 mutation (R493X), the most common *GRN* mutation found in patients with FTD (Table 2). This mutation results in a premature stop codon causing the loss of three amino acids of granulin D and the entire granulin E [128]. Consequently, the mRNA levels are reduced by over 90% in all tissues of ${\mathrm{Grn}}^{R493X/R493X}\;\text{mice}$, and the truncated PGRN protein (∼54 kDa) is not detected in plasma or tissues [128]. ${\mathrm{Grn}}^{R493X/R493X}\;\text{mice}$ exhibit compulsive grooming, and had a shorter median lifespan (575 days) compared to *Grn* ^*+/+*^ mice (816 days) [128]. *Grn*‐cKO mice, generated by deletion of exon 2–4, shows motor impairment as early as 4 months [129]. More than half of these mice died by 23.5 months [130]. PGRN‐deficient mice, generated by deletion of exon 1–4, display normal motor function, but their spatial learning and memory declined by 12 months (Table 2) [131]. PGRN‐deficient mice also develop Gaucher disease (GD)‐like phenotypes, such as hepatosplenomegaly and glycolipid accumulation in bone marrow. These phenotypes can be rescued by supplementation with a human GBA analog [132].

### 2.11 CLN12

The CLN12 subtype is caused by mutations in ATPase Cation Transporting13A2(*ATP13A2*). *ATP13A2* mutations are also associated with Kufor–Rakeb syndrome (KRS), a rare form of autosomal‐recessive hereditary parkinsonism with dementia [133, 134]. KRS is characterized by juvenile‐onset PD with symptoms such as rigidity, spasm, bradykinesia, and cognitive impairment. In contrast, CLN12 disease typically presents with motor dysfunction and cognitive decline [133, 134, 135]. Patients with CLN12 disease generally begin to show gait disturbance around age 13, followed by dysphagia, motor dysfunction, and cognitive impairment in their 20s, with premature death typically occurring around age 40 (Table 1) [134, 135, 136].

ATP13A2 (also known as PARK9) is a transmembrane protein located in the membrane of lysosomes and late endosomes. It functions as a transporter in the late endolysosomal pathway, regulating ion homeostasis (Ca^2+^, Mn^2+^, Zn^2+^, etc.), lipid homeostasis, and the autophagy‐lysosomal pathway [137, 138, 139, 140, 141, 142, 143]. High concentrations of polyamines could induce cell toxicity. ATP13A2 promotes the cellular uptake of polyamines via endocytosis and transports them into the cytosol, while loss of ATP13A2 exacerbates the cell toxicity of polyamines [143].

PD is characterized by the presence of Lewy bodies, which are caused by the accumulation of α‐synuclein, and is also associated with lysosomal and autophagic dysfunction. Loss of ATP13A2 impairs the degradation of lysosomal substrates, reduces lysosome‐mediated clearance of autophagosomes, and induces misfolding and accumulation of α‐synuclein [138, 139, 144]. Additionally, loss of ATP13A2 could reduce the amount and activity of CTSD in the brain [112]. $A\mathrm{tp}1{\text{3a2}}^{-/-}$ mice, generated by replacing exons 12–15 and part of exon 16 with a neo cassette, show comparable sensorimotor function to wild‐type mice at 5–12 months of age. $A\mathrm{tp}1{\text{3a2}}^{-/-}$ mice do not exhibit significant impairments in motor function till 20 months of age (Table 2) [145].

### 2.12 CLN13

CLN13 disease, also known as Kufs disease type B, is caused by mutations in CTSF [146]. Patients with CLN13 disease develop progressive dementia after age 20, with most dying around age 60 (Table 1) [11, 147].

CTSF is a widely expressed lysosomal cysteine protease, which is responsible for the lysosomal processing of lysosomal integral membrane protein type‐2 (LIMP‐2). LIMP‐2 is located in the membrane of lysosomes and has been identified as an M6P‐independent lysosomal transport receptor for β‐glucocerebrosidase (GC) [148]. CTSF deficits are unable to cleave LIMP‐2, causing the lipid metabolism disorders [149]. CTSF and MFSD8 (CLN7) are involved in the lysosomal–autosomal pathway that contributes to amyotrophic lateral sclerosis (ALS)/FTD [150]. CTSF is also associated with tumor growth, invasion, and metastasis, which is identified as a marker of cervical and gastric cancers [151]. CTSF regulates BCL2 expression, and the dysfunction of CTSF promote the proliferation of gastric cancer cells [151, 152].

*Ctsf* ^*−/−*^ mice is generated by deletion of exons 7–9 of the *Ctsf* (*Cln13*) gene. These mice exhibit tonic hind leg extension, poor balance, tremors, spasms, and seizures [49]. Most *Ctsf* ^*−/−*^ mice die about 18 months (Table 2) [49].

### 2.13 CLN14

CLN14 disease is caused by a mutation in potassium channel tetramerization domain‐containing 7 (*KCTD7*). Mutation in *KCTD7* is associated with progressive myoclonic epilepsy type 3 (EPM3) [153, 154, 155, 156]. In 2012, *KCTD7* mutation‐related diseases were defined as CLN14 [156]. Patients with *KCTD7* mutation experience seizure beginning at 14 months of age, followed by developmental delays, with most patients dying between ages 1.5–21 (Table 1) [153].

KCTD7 is a member of the BTB domain‐containing adaptor subfamily. KCTD7 is an adaptor of the CUL3‐RING E3 ubiquitin ligase (CRL3) complex, which recruits and degrades CLN5. KCTD7 mutations disrupt the interaction between KCTD7 and CUL3, leading to excessive accumulation of CLN5 [154]. Disruption of the interaction between CLN6/8 and LEs in the ER impairs ER‐to‐Golgi trafficking of LEs [154]. Calpains, a class of cytosolic calcium‐dependent proteases, regulate cell proliferation, migration, apoptosis, and membrane fusion [157, 158] The KCTD7‐CUL3 ubiquitin ligase regulates calpain activity through n ubiquitination at specific sites. Loss of KCTD7 leads to calpain hyperactivation, causing aberrant cleavage of downstream targets and consequently cell death [158].

*Kctd7*‐deficient mice were generated by the deletion of exon 2. These mice exhibited seizures and myoclonic jerks at 2 months of age and displayed defects in gait at of the same age, characterized by decreased print area, swing speed, and speed for each of the four paws (Table 2) [158, 159].

### 2.14 Subtypes Not Yet Determined

Other genes involved in lysosomal function and their potential link to NCL are continually being explored. These include genes related to the trafficking and degradation of lysosomal substrates, which are critical for maintaining cellular homeostasis and preventing the accumulation of toxic materials within cells. CLN15 has been described and tentatively named in 2018. This subtype is caused by homozygous mutation of the TBC1 domain‐containing kinase (*TBCK*) gene. This disease is characterized by infantile hypotonia with psychomotor retardation and characteristic facial features, known as IHPRF3 [160, 161]. Patients with CLN15 disease develop hypotonia before age 1, followed by intellectual disability, delayed speech, blindness, and seizures. Some patients succumb to the disease around age 10 [160, 161, 162]. Mutation in *TBCK* has been reported to lead to the accumulation of lipofuscin in neurons, leading to neuron loss in various brain regions [160, 162].

In addition, mutations in N‐Sulfoglucosamine Sulfohydrolase (*SGSH*, sulfamidase), typically associated with mucopolysaccharidosis type IIIA (MPSIIIA), have been reported in a case diagnosed with ANCL [11, 163]. Chloride Voltage‐Gated Channel 6 (ClC‐6, CLCN6) has been identified as a potential candidate for mild forms of human NCL. *CLCN6* encodes a Cl^−^/H^+^ exchanger that is predominantly found in late endosomes of nervous system. Moreover, *Clcn6* KO mice display some features of NCL, but without neurodegeneration [164, 165]. A more convincing link between *CLCN6* mutations and human NCL has to be established.

## 3 Therapeutic Strategies for NCLs

Despite the diverse etiologies and initial symptoms, significant strides have been made in developing therapeutic approaches that appear effective across multiple forms of neuronal ceroid lipofuscinoses (NCLs). These innovative treatments include ERT, pharmacological approaches, gene therapy, and hematopoietic stem cell gene therapy. It is crucial to emphasize, however, that for any NCL treatment to be effective in the long term, it must ideally be initiated early, preferably before the onset of neurodegeneration.

### 3.1 Enzyme Rreplacement Ttherapy

Enzyme replacement therapy (ERT) is the most clinically employed approach for LSDs, which is practiced by administration of the missed or defective enzyme systemically [15, 166]. The majority of LEs are directed to the lysosome via the M6P‐dependent pathway. Within the cis‐Golgi apparatus, GlcNAc‐ phosphotransferase appends GlcNAc‐1‐phosphate to high mannose glycans on LEs, a process that occurs concurrently with mannosidase trimming. In the TGN, the uncovering enzyme removes the outer GlcNAc to generate M6P. M6P receptors (M6PRs) on the TGN membrane recognize M6P glycans on LEs, facilitating the transport of the LE‐M6PR complex to the early endosome via clathrin‐coated vesicles. The LE‐M6PR complex dissociates in the acidic environment of the late endosomes, including the multivesicular body (MVB). The released LEs proceed to the lysosome, whereas the M6PRs are recycled back to the TGN through the recycling endosomes. Extracellular proteins containing M6P glycans, such as LEs and ERT enzymes, can be internalized by CI‐M6PR at the plasma membrane through clathrin‐mediated endocytosis and delivered to lysosomes via the late endosomes [167]. ERT is particularly suitable for NCL subtypes resulting from mutations in soluble LEs, including CLN1, CLN2, CLN5, CLN10, and CLN13. However, it is not appropriate for NCLs associated with mutations in lysosomal membrane proteins (CLN3, CLN7, and CLN12), ER membrane proteins (CLN6 and CLN8), or cytosolic proteins (CLN4 and CLN14). Clinical trials assessing ERT treatment for NCL are summarized in Table 3.

**TABLE 3 |:** Clinical trials for treatment of neuronal ceroid lipofuscinoses.

Disease name	Study title	NCT number^a^	Treatment	Phase and status	Patients number	Location	Outcome
CLN1	Cystagon to treat infantile NCL	NCT00028262	Cysteamine bitartrate and N‐acetylcysteine	4, published [168]	10	USA	The therapy is associated with delay of isoelectric EEG, depletion of GRODs, and subjective benefits as reported by parents and physicians. No treatment‐related adverse events occurred apart from mild gastrointestinal discomfort in two patients, which disappeared when liquid cysteamine bitartrate was replaced with capsules
CLN1/2	Study of HuCNS‐SC cells in patients with infantile or late‐infantile NCL	NCT00337636	Human CNS stem cells	1, published [169]	6	USA	Cell transplantation was well tolerated. Observations regarding efficacy of the intervention were limited by the enrollment criteria requiring that patients be in advanced stages of disease
CLN1/2	Safety and efficacy study of HuCNS‐SC in subjects with NCL	NCT01238315	Human CNS stem cells	1, withdrawn (Lack of timely patient accrual)	0	USA	NA
CLN2	A phase 1/2 open‐label dose‐escalation study to evaluate safety, tolerability, pharmacokinetics, and efficacy of intracerebroventricular BMN 190 in patients with late‐infantile neuronal CLN2 disease	NCT01907087	rhTPP1 (cerliponase alfa, BMN190)	1/2, published [170]	24	USA, Germany, Italy, UK	Therapy resulted in less decline in motor and language function than that in historical controls. Serious adverse events included failure of the intraventricular device and device‐related infections The therapy was approved by the FDA and EMA
CLN2	A multicenter, multinational, extension study to evaluate the long‐term efficacy and safety of BMN 190 in patients with CLN2 disease	NCT02485899	rhTPP1 (cerliponase alfa, BMN190)	1/2, completed	23	USA, Germany, Italy, UK	NA
CLN2	A safety, tolerability, and efficacy study of intracerebroventricular BMN 190 in pediatric patients < 18 years of age with CLN2 disease, NCT02678689	NCT02678689	rhTPP1 (cerliponase alfa, BMN190)	2, completed	14	USA, Germany, Italy, UK	NA
CLN2	Safety study of a gene transfer vector for children with late‐infantile neuronal CLN2	NCT00151216	AAV2 CU h‐CLN2 vector	1, published [171]	10	USA	Assessment of the neurologic rating scale demonstrated a significantly reduced rate of decline compared with control subjects. Four of the 10 subjects developed a mild, mostly transient, humoral response to the vector. One subject died 49 days postsurgery after developing status epilepticus on day 14
CLN2	Safety study of a gene transfer vector (Rh.10) for children with late‐infantile neuronal ceroid lipofuscinosis (LINCL)	NCT01161576	AAVrh.10CUhCLN2 vector	1, completed	12	USA	NA
CLN2	AAVRh.10 administered to children with late‐infantile NCL	NCT01414985	AAVrh.10CUCLN2	1/2, completed	8	USA	NA
CLN2	A first‐in‐human study in pediatric patients with ocular CLN2 disease	NCT05791864	RGX 381	1/2, ongoing	16	UK	NA
CLN2	Intravitreal ERT to prevent retinal disease progression in children with CLN2	NCT05152914	rhTPP1 (cerliponase alfa, BMN190)	1/2, ongoing	5	USA	NA
CLN3	Cellcept for treatment of juvenile neuronal ceroid lipofuscinosis (JUMP)	NCT01399047	Mycophenolate mofetil	2, published [172]	19	USA	There were no definite effects on measured autoimmunity or clinical outcomes in the setting of short‐term administration. The most common adverse events did not occur at a significantly increased frequency above placebo
CLN3	Gene therapy for children with CLN3 Batten disease	NCT03770572	scAAV9.P546.CLN3 (AT‐GTX‐502)	1/2, ongoing	7	USA	NA
CLN3	Safety, tolerability, and efficacy of PLX‐200 in patients with CLN3	NCT04637282	PLX200	3, not yet recruiting	39	No location data	NA
CLN3	An open‐label safety, pharmacokinetic, and efficacy study of miglustat for the treatment of CLN3 disease	NCT05174039	Miglustat	1/2, ongoing	6	USA	NA
CLN5	Gene therapy study for children with CLN5 Batten disease (CLN5‐200)	NCT05228145	NGN‐101 (AAV9 carrying the gene encoding human CLN5)	1/2, ongoing	6	USA, UK	NA
CLN6	Gene therapy for children with variant late‐infantile neuronal ceroid lipofuscinosis 6 (vLINCL6) disease	NCT02725580	scAAV9.CB.CLN6 (AT‐GTX‐501)	1/2, completed	13	USA	NA
CLN7	Phase 1 intrathecal lumbar administration of AAV9/CLN7 for treatment of CLN7 disease	NCT04737460	AAV9/CLN7	1, ongoing	4	USA	NA
NCLs & other diseases	Human placental‐derived stem cell transplantation (HPDSC)	NCT01586455	Human placental‐derived stem cell	1, ongoing	43	USA	NA
NCLs & other diseases	UCB transplant of inherited metabolic diseases with administration of intrathecal UCB derived oligodendrocyte‐like cells (DUOC‐01)	NCT02254863	DUOC‐01	1, ongoing	40	USA	NA
NCLs & other diseases	Stem cell transplant for inborn errors of metabolism	NCT00176904	Stem cell transplant	2/3, completed	135 (cases of NCL unknown)	USA	NA

Abbreviations: EMA, European Medicines Agency; FDA, Food and Drug Administration; NCL, neuronal ceroid lipofuscinosis.

^a^ National Clinical Trial (NCT) number obtained from ClinicalTrials.gov.

In 1991, the Food and Drug Administration (FDA) approved Alglucerase as the first ERT for treating GD, a chronic congenital disorder of lipid metabolism caused by a deficiency of the lysosomal enzyme GBA. To date, FDA‐ or European Medicines Agency (EMA)‐approved ERT therapies are available for GD type 1, Fabry disease (FbD), Pompe disease, mucopolysaccharidosis (MPS) 1 (or Hurler syndrome), MPS 2 (or Hunter syndrome), MPS 4 A (or Morquio syndrome), MPS 6 (or Maroteaux‐Lamy syndrome), MPS 7 (or Sly syndrome), α‐mannosidosis (AM) deficiency, lysosomal acid lipase (LAL) deficiency, and CLN2 [166].

In 2012, a US lab reported that purified recombinant PPT1 administration releases the symptoms in *Ppt1* knockout mice, suggesting ERT as a potential treatment to reduce excessive accumulation of lysosomal lipofuscin in the peripheral organs. However, more effective delivery methods to target the brain are deemed necessary [173]. In 2017, human recombinant TPP1 (tripeptidyl peptidase 1; cerliponase alfa) was approved for the treatment of CLN2 through intracerebroventricular (ICV) injection of 300 mg every other week [11, 174, 175]. Regrettably, 18 children with CLN2 disease treated with ERT slowed but did not stop movement disorder progression [176]. In 2020, researchers explored an ERT approach for CLN10 using recombinant human CTSD, demonstrating correction of lysosomal hypertrophy and impaired autophagic flux in CNS and viscera [103]. However, single treatment might not be sufficient to cure NCL, and more than 90% of small‐molecule drugs and nearly 100% of larger‐molecule neurotherapeutics, such as enzymes, cannot cross the blood–brain barrier (BBB) [177, 178]. Therefore, researchers are exploring combinatorial approaches to address this issue. Research efforts are directed toward the development of new strategies to engineer drugs capable of BBB penetration or deliver enzymes to the whole brain [125, 178].

### 3.2 Pharmacological Approaches

Small‐molecule drugs represent a promising targeted approach for addressing rare diseases. Their ability to cross the BBB and reach the CNS makes them a particularly valuable strategy in this context. The development of small‐molecule drugs encompasses both de novo drug development and drug repurposing. De novo development, while targeted, if often costly and time‐consuming. In contrast, drug repurposing offers a more cost‐effective and expedited approach. However, given that a single drug may interact with multiple targets and exert multiple effects, each drug must be evaluated on a case‐by‐case basis [179].

N‐(tert‐Butyl) hydroxylamine (NtBuHA) is a small molecule capable of crossing the BBB and cleaving thioester linkages in palmitoylated proteins. In PPT1‐deficient mice, NtBuHA is shown to deplete lysosomal ceroid, suppress neuronal apoptosis, slow neurological deterioration, and extend the lifespan of PPT1‐deficient mice from 242 to 277 days [180]. Trehalose, another small molecule, activates transcription‐factor EB (TFEB), thereby reducing the accumulation of lipopigments and neuroinflammation in *Cln3* ^*Δex7/8*^ mice. Oral administration of trehalose to *Cln3* ^*Δex7/8*^ mice has been found to extend their lifespan from 454 to 522 days [170]. While studies have demonstrated the neuroprotective effects of trehalose in animal models, the mechanisms underlying its neuroprotective actions and its ability to cross the BBB remain to be elucidated [181, 182]. In a similar vein, flupirtine, which exerts neuroprotective effects through the upregulation of Bcl‐2 and antagonism at the NMDA receptor, was a non‐opioid analgesic previously approved for the treatment of seizures in Europe [183]. Flupirtine has also been shown in vitro to reduce apoptosis in CLN3 lymphocytes [184]. However, its clinical use has been significantly restricted. In 2018, the EMA withdrew approval for flupirtine medication in European countries due to the risk of acute liver failure [185]. Peroxisome proliferator‐activated receptor—alpha (PPARα) agonists have been shown to induce autophagy. Gemfibrozil, an FDA‐approved PPARα agonist used to regulate cholesterol, has been found to decrease cellular accumulation of lipofuscin, improve motor coordination, and extend the lifespan of CLN2‐deficient mice from 120 to 180 days [186]. Oral administration of gemfibrozil has also been shown to preserve the integrity of the BBB and blood–spinal cord barrier, reducing the infiltration of mononuclear cells into the CNS.

Neuroimmune responses mediated by astrocytes and microglia are integral to the progression of neurodegenerative diseases. These cells modulate inflammation through the secretion of cytokines and chemokines, which can either exacerbate or mitigate neurodegeneration. PLX3397, a CSF‐1R inhibitor capable of crossing the BBB, has been shown to effectively eliminate microglia. In PPT1‐deficient mice, treatment with PLX3397 led to a reduction in mRNA expression of pro‐inflammatory cytokines such as IL‐1β, TNFα, and CXCL10, thereby diminishing neuroinflammatory responses, attenuating neurodegeneration, and improving clinical outcomes [187]. Another BBB‐penetrant, anti‐neuroinflammatory agent, MW01–2‐151SRM (MW151), is a small molecule that mitigates glial cytokine upregulation in models of neuroinflammation. When combined with AAV‐mediated gene therapy, MW151 extended the lifespan of PPT1‐deficient mice from 34.9 to 47 weeks, improved motor performance, and eliminated seizures [188].

The upregulation of glial fibrillary acidic protein (GFAP) serves as a classic marker for astrogliosis. PPT1 plays a regulatory role in GFAP depalmitoylation, and in PPT1‐deficient mice, astrocytes exhibit a more activated morphology along with elevated expression levels of GFAP. Notably, the pathogenic role of glial cells may differ among different NCL subtypes. For example, in CLN3‐deficient mice, the hypertrophy of astrocyte cell bodies and processes, as well as GFAP upregulation, appears to be more subtle or perhaps attenuated compared to the pronounced changes observed in astrocytes from PPT1‐deficient mice [189, 190, 191]. Ibuprofen, an anti‐inflammatory, and lamotrigine, an anticonvulsant with neuroprotective properties, have been shown to improve certain motor skills in CLN3‐deficient mice when used in combination. This combined treatment has moderate effects on astrocyte activation (especially ibuprofen) but does not significantly impact microglial activation [192].

Other anti‐inflammatories include Fingolimod (or FTY720), a sphingosine‐1‐phosphate (S1P) receptor modulator that impairs lymphocyte emigration from secondary lymphatic organs and infiltration into the CNS; Teriflunomide, a selective dihydroorotate dehydrogenase inhibitor, attenuates the proliferation and mitochondrial respiration of activated, high‐affinity lymphocytes. Both of these drugs have been shown to reduce CNS inflammation, improve motor coordination, and enhance visual acuity in PPT1‐deficient mice [193]. It's worth mentioning that anti‐inflammatory agents may be most effective when used in conjunction with other treatments [194]. Demyelination, a pathological change closely associated with oligodendrocytes, is prevalent in numerous neurodegenerative diseases, including CLN1, CLN5, CLN8, and CLN11 [189, 195]. While drugs targeting demyelination hold promise, the body of research substantiating their efficacy remains limited.

The field of RNA therapeutics has been remarkable progress since 2013. Structurally, RNA therapeutics can be categorized into ASOs, small interfering RNAs (siRNAs), mRNA, and aptamers. The majority of RNA therapeutics that have gained approval to date are ASOs and siRNAs. As of 2024, 10 ASOs, 6 siRNAs, and one single aptamer have been commercialized. ASOs are comprised of short, single‐stranded nucleic acids, typically 15–30 bases in length. In contrast, siRNAs are characterized by their well‐defined structure, consisting of short RNA duplexes (usually 20–24 base pairs) with two‐base overhangs in the 3′ region [196, 197, 198].

The pharmacokinetic profile of ASO drugs, encompassing absorption, distribution, metabolism, and excretion (ADME), diverges significantly from that of small‐molecule drugs. The ADME characteristics of small‐molecule drugs are typically governed by uptake and efflux transporters, phase I and II drug‐metabolizing enzymes, transcription factors, and xenobiotic‐activated nuclear receptors. In contrast, the absorption of ASOs into target cells is facilitated through endocytic internalization into early endosomes, followed by subcellular trafficking within the cell. The release of ASOs from endosomes is a critical step that influences both therapeutic efficacy and the potential for adverse drug reactions [197]. Drug–drug interactions are infrequent with ASOs, as their metabolism does not involve the traditional drug‐metabolizing enzymes or transporters [196]. The FDA‐approved ASO drugs are administered via four routes: intravitreal injection, intrathecal injection, subcutaneous injection, and intravenous infusion (i.v.), ASO administered intrathecally to humans or nonhuman primates are distributed to the brain with approximately one‐third the efficiency of their distribution to the spinal cord [197, 199]. Unlike small‐molecule drugs, which often have a short half‐life necessitating daily dosing due to oral administration, ASO drugs exhibit a longer half‐life. This extended half‐life allows for less frequent dosing requirements, such as weekly or monthly administrations [197].

*Cln3* ^*Δex5/7/8*^ mice, which lack exons 5, 7, and 8, exhibit a reduced disease burden both behaviorally and pathologically when compared with ${\text{Cln3}}^{\mathrm{\Delta ex}7/8}$ mice. This reduction is more pronounced in homozygous *Cln3* ^*Δex5/7/8*^ mice than in those with only one copy of *Cln3* ^*Δex5/7/8*^ allele, suggesting a dose‐dependent relationship between the presence of *Cln3* ^*Δex5/7/8*^ and therapeutic outcomes. Therapeutic efficacy may be achieved with ≥50% skipping of exon 5 in ${\text{Cln3}}^{\mathrm{\Delta ex}7/8}$ mice [34]. Splice‐switching antisense oligonucleotides (SSOs) are a class of ASO that function by base‐pairing to their target RNA, effectively concealing it from the RNA processing machinery. SSO can specifically target endogenous pre‐mRNA splicing and exclude (skip) exons containing the PTCs [34]. An SSO (ASO‐26) designed to induce robust exon 5, an alternatively spliced exon, has the potential to restore the open reading frame of ${\text{Cln3}}^{\mathrm{\Delta ex}7/8}\;\text{mice}$. Homozygous ${\text{Cln3}}^{\mathrm{\Delta ex}7/8}\;\text{mice}$ treated on postnatal day 1 or 2 (P1–2) with ICV injection of ASO‐26 demonstrate improved motor function on the rotarod test and an extended lifespan of ${\text{Cln3}}^{\mathrm{\Delta ex}7/8}$; hAPP mice from 18.5 to 53 days [200].

In a case involving a patient with CLN7 disease, the retrotransposon insertion resulted in the aberrant splicing of exon 6 to a cryptic splice‐acceptor site (i6.SA) within the MFSD8* intron 6, likely leading to premature translational termination. To address this, investigators developed a customized ASO, Milasen, designed to target the i6.SA cryptic splice‐acceptor site and adjacent splicing enhancers. Following a 1‐month toxicologic evaluation and securing authorization from the FDA along with expedited institutional review board approval, the investigators administered intrathecal administration of Milasen to the patient, starting at 3.5 mg and incrementally increasing approximately every 2 weeks up to 42 mg. Throughout the first year of treatment, no serious adverse events occurred, and there was a > 50% reduction in both the frequency and duration of seizures [199]. However, it is important to note that the design of Milasen is customized especially for this patient's unique mutation and is not applicable for the treatment of other patients with Batten disease. Considering manufacturing capacity and cost, this approach is likely scalable to only a limited number of patients at present [168, 199].

In addition to the pharmacologic treatments discussed above, many current pharmacological approaches for NCLs primarily focus on clinical symptoms such as seizures, rather than targeting the underlying disease mechanisms. However, follow‐up studies using these drugs have thus far failed to demonstrate clinically meaningful benefits for patients [11]. Despite these limitations, we believe that emerging or future drug discovery targets for NCLs have the potential to address these shortcomings, or they will probably be used to supplement other treatments and methods such as gene therapy.

### 3.3 Gene Therapy

Gene therapy presents an alternative therapeutic approach for NCLs associated with mutations in lysosomal membrane proteins, ER membrane proteins, or cytosolic proteins. Additionally, it is a viable option for NCLs resulting from mutations in soluble LEs. In recent years, gene therapy has gained increasing attention as a potential treatment for NCL. Clinical trials assessing gene therapy for NCL are summarized in Table 3.

For CLN2 disease, the latest therapeutic strategy is intracisternal magna (ICM)‐delivered adeno‐associated vector (AAV) gene therapy. While intraparenchymal (IPC) administration of AAVrh.10hCLN2 exhibited therapeutic benefits over 18 months, it did not halt disease progression, indicating the need for alternative administration routes to enhance therapeutic efficacy [169]. ICM injection of AAVrh.10hCLN2, encoding human CLN2, at a dose of 5 × 10^13^ genome copies (gc) per nonhuman primates (NHPs), facilitates TPP1 expression. TPP1 diffusion into CSF and throughout the brain was observed, suggesting potential therapeutic benefits [171].

ICV injection of scAAV9.Mecp2.CLN3 at a dose of 2.2 × 10^10^ vector genome (vg) per mouse at postnatal day 0 (P0) or P1 overexpresses human CLN3 (hCLN3) protein throughout the CNS [172]. This partially alleviates motor defects of ${\text{Cln3}}^{\mathrm{\Delta ex}7/8}$ mice, but does not improve learning and memory impairment or visual failure [172]. This therapeutic strategy prompted the launch of a first in‐human clinical trial (NCT03770572, Table 3) [172]. Besides, ICV delivery of scAAV9/oCLN5 at a dose of 2.8 × 10^12^ vg per sheep at 6–7 months of age extends the lifespan from 20 to 60 months, but does not preserve eyesight [201]. One first‐in‐human open‐label, dose‐escalation study designed to assess the safety and efficacy of the administration of an AAV9 carrying the gene encoding human CLN5 in subjects with CLN5 disease has started since 2022 (NCT05228145, Table 3).

Intrathecal (IT) delivery of AAV9/MFSD8 at a dose of 5 × 10^11^ vg per mouse at P7–P10 could extend the lifespan of *Cln7* KO mice from 8 to 17 months [88]. ICV delivery of scAAV9.pT‐MecP2.CLN8 at a dose of 5 × 10^10^ vg per mouse at P1 could relieve motor defects and extend the lifespan of *Cln8* ^*mnd*^ mice from 10 months to over 24 months [202]. Intravitreal injection of scAAVshH10‐CTSD at a dose of 2.5 × 10^12^ vg per mouse at P5 could attenuate retinal degeneration and rescue photoreceptor cells and rod bipolar cells [203]. Intravitreal injection of AAV2.7 m8‐scCAG‐PGRN at 7.94 × 10^10^ vg per mouse at 1, 6, and 12 months of age; IV injection of AAV9.2YF‐scCAG‐PGRN at 1.2 × 10^12^ vg per mouse at P3–P4; and ICV delivery of AAVhu68 at 1× 10^11^ genomic copies (GC) per mouse at 2 months of age could reduce lipofuscin deposits in the CNS of GRN KO mice [204, 205].

An ongoing clinical trial for CLN6 disease is ICV‐delivered self‐complementary AAV9 (scAAV9) gene therapy (NCT02725580, Table 3). In *CLN6* ^*nclf*^ mice, ICV delivery of scAAV9.CB.CLN6 at a dose of 5 × 10^10^ vg per mouse at P1 overexpresses CLN6 throughout the CNS [72]. This significantly alleviates motor defects, delays learning and memory impairment, and extends lifespan from approximately 14 to 24 months [72]. Besides, it prevents Batten disease pathology in the brain's visual processing centers, preserving neurons and partially preserved visual acuity in *Cln6* ^*nclf*^ mice [206].

### 3.4 Hematopoietic Stem Cell Gene Therapy (HSC‐GT)

Hematopoietic stem cell therapy (HSCT) has been proposed for several LSDs; for example, in MPS 1, HSTC has shown to increase life expectancy and improve some clinical manifestations [207]. However, HSCT was proven to be ineffective in a number of trials of enzyme‐deficient forms of NCL about 20 years ago [208, 209, 210, 211]. In recent years, HSC‐GT has shown good therapeutic effects in mouse model of CLN1 disease (Table 3). Overexpressing PPT1 on hematopoietic stem cells by infection of lentivirus (LV), which are then delivered intravenously (IV), has been shown to extend the lifespan of CLN1‐deficient mice from approximately 250 days to over 350 days [14]. However, achieving sufficient transplantation efficiency in the brain remains a challenge due to ineffective microglial removal following busulfan‐induced BBB opening [14]. Current clinical trials assessing HST‐GT for NCL are summarized in Table 3.

Microglia are prominent immune cells in CNS. Microglia originate from myeloid hematopoietic stem cells, like peripheral macrophages; they possess a variety of myeloid cell properties [212]. Microglia constantly monitor the environment, phagocytose damaged cells, extracellular matrix, debris of cells and myelin, invading pathogens, etc. In addition to immune functions, microglia play important roles in maintaining healthy neural circuits and regulating synaptic plasticity. Microglia release substances that can affect neurons, in CNS, they regulate proliferation and migration of neuronal precursors, neuronal differentiation, axon growth, myelin sheath formation, synaptogenesis and synaptic pruning [213, 214]. In adult mammalian brain, microglia are regenerated by self‐renew, coupled with cellular senescence and death to maintain a stable cell population. Under physiological conditions, microglia turnover at a median rate of 28% per year. Under pathological conditions, rapid microglia turnover is observed [215]. In CNS, colony‐stimulating factor 1 receptor (CSF1R) is specifically expressed on microglia membranes. Microglia differentiation and survival are dependent on the CSF1 signaling pathway. Knockdown or specific inhibition of CSF1R completely eliminates microglia from the brain; when inhibition is stopped and after a brief recovery period, newborn microglia appear in the brain. These regenerated microglia arise from self‐proliferation of endogenous microglia, can rapidly colonize the entire brain and return to their original state, a process known as microglia repopulation [216].

HSC‐derived cells are able to differentiate into microglia and act as a constant source of enzyme secretion and have a supportive influence in the CNS. When neurons of *Cln3* ^*Δex1–6*^ mice were co‐cultured with WT astrocytes and microglia, the defects found in mutant neurons could be markedly improved [191]. Microglia are abundant throughout the brain and represent a promising target for precise gene correction to alleviate NCL pathology [214, 217]. AAV and retrovirus are the most commonly used vehicles for gene delivery, though historically considered ineffective for targeting microglia [218]. Recently, an AAV variant showed efficient transduction of microglia in vivo following IV delivery, achieving up to 80% transgene expression. This result has yet to be verified [219, 220, 221]. LV are capable of transducing HSC and HSC‐derived cells (including microglia) with high efficiency, so it is generally utilized in HSC‐GT [222]. In addition to virus‐based gene therapies, replacing endogenous microglia with foreign cells is another potential strategy to delivery enzymes to the whole brain. However, the efficiency of HSCT in the CNS is not high, which limits the use of HSCT in the NCL. Traditional bone marrow transplantation (tBMT) has shown limited success due to low replacement efficiency (usually < 5%–20%) [223, 224]. In contrast, microglia replacement by bone marrow transplantation (Mr BMT) was developed in 2020, achieving high efficiency in mice with CNS‐wide replacement of resident microglia (92.66% in the brain, 99.46% in the retina, and 92.61% in the spinal cord) [225]. Targeting microglia for NCL treatment, especially through Mr. BMT combined with gene therapy, offers a promising therapeutic approach.

## 4 Conclusions and Perspectives

The pathogenic mechanisms, age of onset, and symptomatic presentations of various NCLs resulting from mutations in diverse genes vary significantly. However, most clinical manifestations are characterized by seizures, visual deficits, and motor and cognitive decline. The rarity of NCL in clinical settings and its severe consequences limit therapeutic options, placing significant stress on patients and families.

Fortunately, recent years have seen expanded understanding of NCL diseases through the correlation of genotypes with clinical phenotypes. This correlation has facilitated attention to gene‐based targets essential for therapeutic advancements. Although these therapies are currently in early stages of development, many hold promise as potentially disease‐modifying, capable of slowing or halting disease progression. Unfortunately, these therapies are unlikely to achieve partial disease reversal, and complete reversal remains improbable. Thus, with the advent of these damage‐limiting interventions, early diagnosis and treatment are more crucial than ever. Looking forward, it is anticipated that combining multiple treatments effectively will yield improved outcomes, offering hope to patients and families alike.

## Conflicts of Interest

The authors declare no conflicts of interest.

## Data Availability

Data sharing is not applicable to this article as no new data were created or analyzed in this study.
